# Bullous Systemic Lupus Erythematosus As the Initial Presentation of Systemic Lupus Erythematosus

**DOI:** 10.7759/cureus.93114

**Published:** 2025-09-24

**Authors:** Cholponai Kydyralieva

**Affiliations:** 1 Pathology, Kyrgyz State Medical Academy, Bishkek, KGZ

**Keywords:** autoimmune blistering disease, bullous systemic lupus erythematosus, dapsone therapy, subepidermal blisters, systemic lupus erythematosis

## Abstract

Bullous systemic lupus erythematosus (BSLE) is a rare, autoantibody-mediated subepidermal blistering disorder. It typically occurs in patients with established systemic lupus erythematosus (SLE). We report a 55-year-old Hispanic female with no prior SLE history who developed bullous lesions later identified as BSLE. Skin biopsy and direct immunofluorescence showed subepidermal blisters, and these findings, along with her clinical presentation, confirmed the diagnosis. Linear deposition of IgG, complement C3, and collagen VII antibodies was found, which is highly characteristic and supportive of BSLE. In this clinical context, these immunopathologic findings are considered diagnostic; however, confirmation can be further supported by a rapid response to dapsone therapy. Oral dapsone therapy resulted in complete resolution within weeks. No residual scarring or pigmentation remained. This case highlights the importance of considering BSLE even in patients without classical lupus features.

## Introduction

Bullous systemic lupus erythematosus (BSLE) is a rare autoimmune blistering disorder characterized by the rapid onset of vesiculobullous lesions, most commonly affecting sun-exposed areas [1]. The reported incidence of BSLE among patients with systemic lupus erythematosus (SLE) is less than 5%, with published rates ranging from 0.19% to 5% [1-3]. Presentation of BSLE as the initial manifestation of lupus is exceedingly uncommon and not well-documented in the literature. Diagnosing BSLE can be challenging because its features overlap with other autoimmune blistering diseases, such as epidermolysis bullosa acquisita (EBA), bullous pemphigoid (BP), dermatitis herpetiformis (DH), and linear IgA bullous dermatosis [4,5]. Furthermore, patients with BSLE rarely exhibit the classic cutaneous lesions seen in discoid, systemic, or subacute cutaneous lupus erythematosus, further complicating diagnosis [6].

Early recognition of BSLE is clinically important, as timely diagnosis can prevent unnecessary immunosuppression and allow for rapid initiation of effective treatment, such as dapsone.

Here, we present a rare case of a 55-year-old Hispanic female with no prior history of SLE who developed bullous lesions as the initial manifestation of BSLE, underscoring the need for awareness of this atypical presentation in clinical practice.

## Case presentation

A 55-year-old Hispanic female with a history of Hashimoto's thyroiditis, managed with levothyroxine, presented to our dermatology clinic with erythema, crusting, and areas of hardening or scarring localized to her forehead. The lesions had gradually progressed over several weeks, accompanied by mild pruritus but no systemic symptoms such as fever, joint pain, or other signs suggestive of lupus. She had no personal or family history of SLE. Initial treatment with triamcinolone acetonide 0.025% ointment was ineffective.

**Figure 1:**
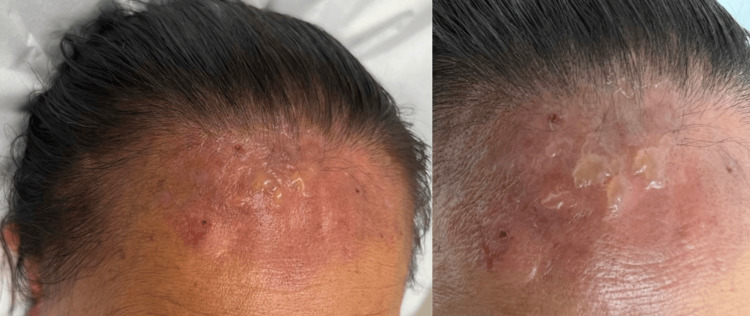
Multiple tense bullae on an erythematous base on the forehead, ranging from 0.5 to 2 cm in diameter, some with crusted surfaces from rupture.

Several weeks later, the patient returned with more extensive findings. Examination revealed multiple tense, round-to-oval bullae (1-2 cm) filled with serous fluid on an erythematous base, surrounded by dryness and scaling. Areas of erosion and yellowish, crusted plaques were present where bullae had ruptured (Figure 1). Lesions were confined to sun-exposed skin, without mucosal involvement. She reported no recent infections, new medications, or other triggers aside from three prior COVID-19 vaccinations.

An autoimmune panel revealed positive Sm/RNP antibodies. All other major markers, including ANA, anti-dsDNA, anti-Ro/SSA, anti-La/SSB, anti-Smith, and anti-phospholipid antibodies, were negative. The combination of bullous lesions and positive Sm/RNP antibody raised suspicion for BSLE, even in the absence of classic lupus symptoms or a history of SLE. A skin biopsy with direct immunofluorescence (DIF) was performed for further evaluation.

**Figure 2:**
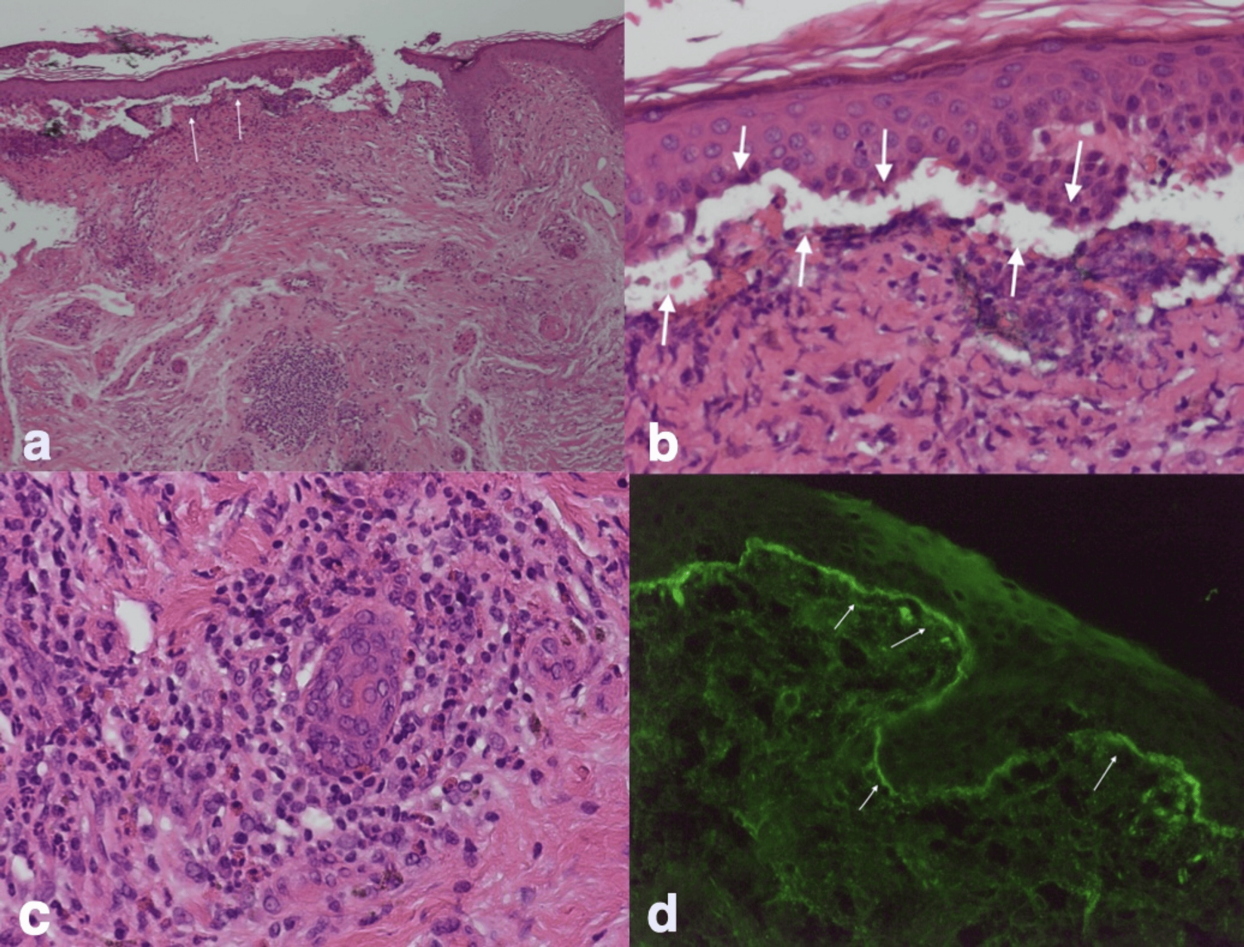
Microscopic appearance of bullous skin lesion. (a) H&E stain. Low-power view showing subepidermal blister formation with separation of the epidermis from the dermis. (b) H&E stain. Higher-power view highlighting separation at the basement membrane zone with neutrophilic infiltration concentrated in the dermal papillae. (c) H&E stain. Neutrophilic infiltration at the dermal papillae, with scattered eosinophils and dermal edema. (d) Direct immunofluorescence demonstrating strong linear deposition of IgG, C3, and collagen VII at the dermo-epidermal junction.

Punch biopsy demonstrated subepidermal blister formation with a neutrophilic infiltrate concentrated in the dermal papillae. Hematoxylin and eosin staining revealed separation of the epidermis from the dermis at the basement membrane zone (BMZ), with numerous neutrophils, eosinophils, and dermal edema (Figures 2a-2c). DIF showed strong linear deposition of IgG, complement C3, and collagen VII at the dermo-epidermal junction, confirming BSLE (Figure 2d).

**Figure 3:**
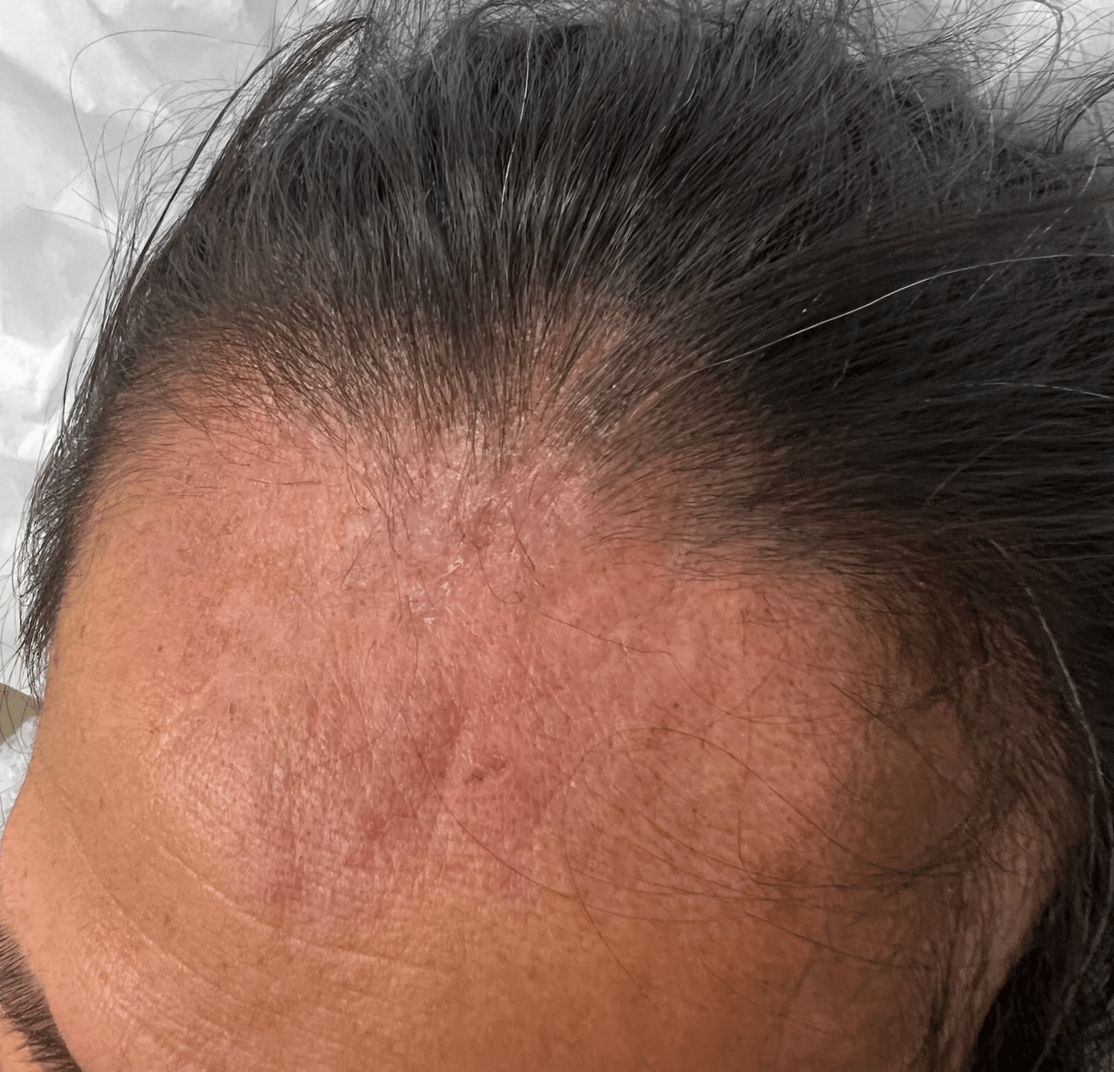
Healed lesions without residual scarring or pigmentation.

After confirming normal CBC and G6PD levels, oral dapsone was initiated at 50 mg twice daily and increased to 100 mg daily after a favorable initial response. The lesions resolved within weeks, leaving no residual scarring or pigmentation (Figure 3). The patient was regularly monitored for clinical improvement and potential dapsone-related adverse effects, such as hemolytic anemia.

## Discussion

BSLE is a rare autoimmune blistering disorder that typically develops in the context of SLE [1-2]. However, in rare instances, it may present in patients without an established SLE diagnosis, which complicates recognition and management [7]. Our case highlights this atypical scenario.

The differential diagnosis in patients presenting with vesiculobullous lesions includes BP, EBA, linear IgA bullous dermatosis, and DH. BP typically occurs in older adults, presents with tense bullae, and demonstrates linear IgG and C3 deposition at the BMZ but lacks collagen VII involvement. EBA may closely resemble BSLE but frequently heals with scarring and milia and is characterized by anti-collagen VII antibodies. DH presents with grouped vesicles, often on extensor surfaces, and shows granular IgA deposits in the dermal papillae, while linear IgA bullous dermatosis reveals linear IgA deposition at the basement membrane [2,4,5,8,9].

In our patient, the absence of systemic lupus features and negative ANA/anti-dsDNA initially delayed consideration of BSLE. Topical corticosteroid therapy was attempted, but proved ineffective. A confirmatory biopsy with DIF was ultimately required to establish the diagnosis. Histology revealed subepidermal blister formation with neutrophilic infiltration of the dermal papillae, and DIF demonstrated strong linear deposition of IgG, C3, and collagen VII along the BMZ, findings characteristic of BSLE [2,5,8]. The autoimmune panel highlighted a positive Sm/RNP antibody, while ANA, anti-dsDNA, anti-Ro/SSA, anti-La/SSB, anti-Smith, and antiphospholipid antibodies were negative. Taken together, these results, combined with the clinical presentation and exclusion of other bullous diseases, established the diagnosis of BSLE.

After BSLE was confirmed and baseline CBC and G6PD testing were normal, dapsone therapy was initiated. Dapsone is widely regarded as the first-line treatment for BSLE, with rapid clinical responses reported in up to 90% of cases [4,10]. Our patient demonstrated complete resolution of lesions within weeks, leaving no scarring or pigmentary change, consistent with prior reports. While effective, dapsone requires close laboratory monitoring due to risks of hemolytic anemia [11], methemoglobinemia, and agranulocytosis [12]. In refractory or systemic cases, additional therapies such as corticosteroids, immunosuppressants, or biologics (e.g., rituximab, belimumab) may be required [13-14].

This case underscores several important lessons. First, BSLE should remain in the differential diagnosis of blistering diseases, even in patients lacking systemic lupus markers or history. Second, histology and DIF findings are indispensable for establishing the diagnosis and avoiding misclassification. Third, dapsone, when initiated after appropriate safety evaluation, provides rapid and effective control of disease while minimizing unnecessary immunosuppression.

## Conclusions

This case contributes to the growing body of literature on BSLE as an initial presentation in patients without a history of SLE. BSLE can present atypically in patients without a prior history of SLE. Early biopsy and DIF, along with starting dapsone therapy, can result in rapid lesion resolution. This prevents long-term sequelae. Clinicians should maintain a high index of suspicion for BSLE in patients with vesiculobullous presentations, regardless of their prior SLE history.
